# The emergence of non-infectious epiglottitis after the era of *Hemophilus influenza type B* universal vaccination: two case reports and literature review

**DOI:** 10.3389/fped.2024.1374311

**Published:** 2024-07-04

**Authors:** Alaa Safia, Rabie Shehadeh, Shlomo Merchavy

**Affiliations:** Department of Otolaryngology, Head and Neck Surgery, Ziv Medical Center, Safed, Israel

**Keywords:** epiglottitis, non-infectious, pediatric, children, airway

## Abstract

**Objective:**

Following the global dissemination of vaccination protocols for *Hemophilus influenza type B*, the occurrence rate and burden of infectious epiglottitis decreased rapidly and substantially. However, this gave space to the rise of non-infectious causes, with children being more vulnerable than adults. This report aims to divert the clinician's attention to the existence of other non-infectious causes of epiglottitis, all of which require careful attention and timely management for favorable clinical outcomes.

**Case summary:**

Two cases with positive vaccination history were encountered in our practice. The first case was a 4-year-old girl, presenting with stridor drooling, and dyspnea, later complication by apnea. The fiberoptic exam revealed severe epiglottitic swelling with near-complete injury of the vocal cords with a circumferential burn. She did not respond to racemic epinephrine, and a tracheostomy was done. She was discharged after 6 days with minimal soft palate swelling. The second case was a 2-year-old boy presenting after exposure to an alkaline solution. The case exhibited similar symptoms but with white plaques and edema of the soft palate. The fiberoptic exam showed swelling and erythema of the supraglottic structures with partial obstruction. Although the blood culture was negative, he was intubated and given four intravenous boluses of dexamethasone and penicillin. On the fifth day, the patient was discharged after a normal fiberoptic examination.

**Discussion:**

These cases highlight a crucial shift in the etiology of epiglottitis post widespread *Hemophilus influenza type B* vaccination, underscoring the emergence of non-infectious causes, particularly in children. The two cases presented, both with vaccination histories, demonstrate diverse non-infectious triggers leading to severe epiglottitis. The first case, involving thermal injury, and the second, chemical exposure, both necessitated intensive interventions, including tracheostomy and intubation. These instances emphasize the need for heightened clinical vigilance for non-infectious epiglottitis, demanding prompt recognition and management to ensure positive outcomes. This shift in etiology calls for a reevaluation of traditional diagnostic and therapeutic approaches to pediatric epiglottitis.

## Introduction

Epiglottitis, a rare yet potentially life-threatening condition, has long been considered a medical emergency due to its capacity to cause acute and complete airway obstruction (1). Epiglottitis, once predominantly caused by *Hemophilus influenzae type B* (Hib), has been a significant concern in pediatric medicine due to its rapid onset and potential to cause life-threatening airway obstruction. Prior to the widespread implementation of Hib vaccination, acute epiglottitis in children was primarily infectious, characterized by high fever, severe throat pain, drooling, and a rapid progression to respiratory distress (2). This condition necessitated urgent medical intervention to secure the airway, often through intubation or tracheostomy.

With the introduction and universal adoption of the Hib vaccine, the incidence of infectious epiglottitis has dramatically decreased, leading to a shift in the etiological landscape (3). Presently, non-infectious factors, such as thermal injuries from hot liquids or chemical burns from caustic ingestions, have emerged as significant causes. These cases typically present with similar acute symptoms but differ in etiology, lacking systemic signs of infection like fever. Recognizing these causes is crucial, as the clinical approach and management may vary from infectious forms (4).

In the context of pediatric airway diseases, it is also useful to differentiate epiglottitis from infant croup, which is commonly caused by viral agents and presents with a barking cough, stridor, and hoarseness, but without the severe drooling and dysphagia typically seen in epiglottitis (5). Croup generally involves the subglottic region and is less likely to cause acute severe airway obstruction, making its management considerably different from that of epiglottitis.

The pediatric airway differs notably from that of adults; it is shorter, narrower, and more compliant. These anatomical features mean that even a small degree of inflammation or swelling can rapidly progress to critical airway obstruction (6). This makes prompt recognition and management of epiglottitis crucial to prevent catastrophic outcomes in children (Supplementary Figure S1).

Herein, we present two compelling cases of children who exhibited clinical and radiological signs mimicking acute infectious epiglottitis but were ultimately diagnosed with traumatic epiglottitis stemming from non-infectious causes. Informed consent was retrieved from both cases. The reporting of this case report adhered to the CARE guidelines.

The distinctiveness of our cases lies in their non-infectious etiologies of epiglottitis in vaccinated children, a notable deviation in the post-*Hemophilus influenza type B* vaccine era. One case presented with thermal injury-induced epiglottitis and the other with chemical exposure, both requiring aggressive interventions. These cases highlight a pivotal shift in the understanding of epiglottitis causality, emphasizing the importance of considering non-infectious factors in diagnosis and management, and underscoring the potential severity of such cases in the pediatric population. This contributes uniquely to the evolving landscape of pediatric otolaryngology in the context of widespread vaccination.

## Case description

### Case one

**Figure 1:**
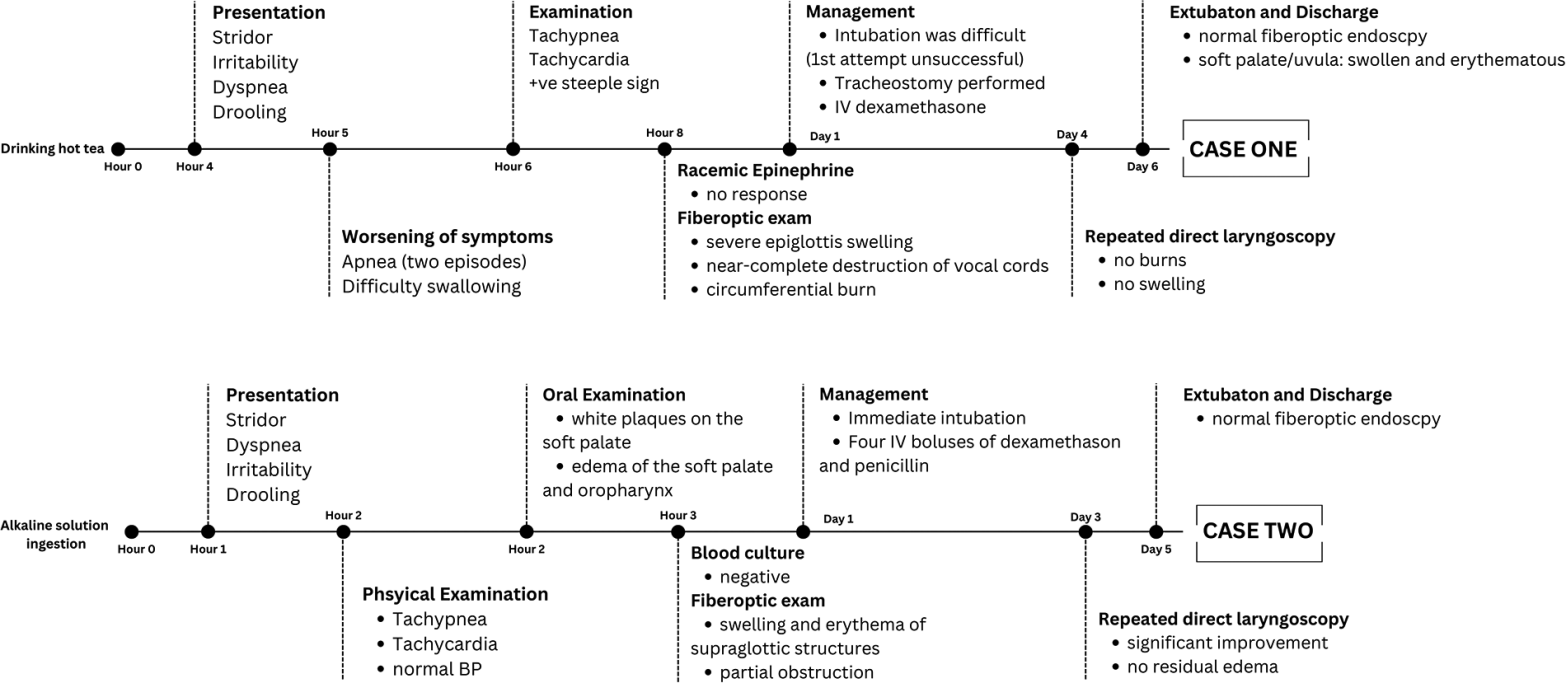
Two cases of non-infectious epiglottitis, 4- and 2-years old children. Paragraph shows the timeline of cases presentation, management, and outcome.

A 4-year-old female from the Upper Galilee region in Israel was admitted to the emergency department after an incident involving swallowing hot tea with a straw. The onset of her symptoms, which included marked irritability, stridor, dyspnea, and drooling, was insidious and began about four hours prior to hospitalization (Figure 1). These symptoms progressively worsened and were accompanied by difficulty in swallowing and two episodes of apnea. Importantly, there was no history of vocal abuse, respiratory infection, cough, fever, surgery, or trauma. The parents reported a positive vaccination history against Hib.

**Figure 2:**
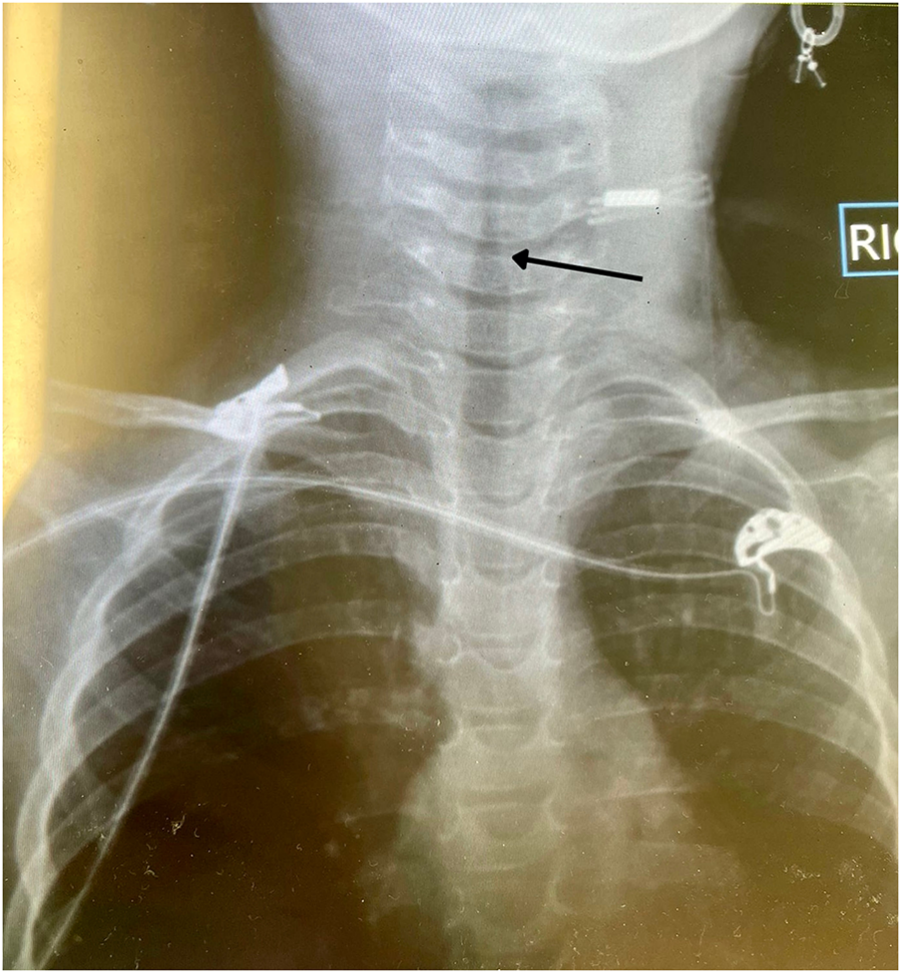
Two years old male with thermal injury-induced epiglottitis. Photograph of lateral neck radiology showing a positive steeple sign.

On examination, she displayed tachypnea and tachycardia, but no external burns or notable findings in the oral and oropharyngeal cavities. A lateral neck radiograph showed a steeple sign, initially suggesting croup (Figure 2). However, after the lack of response to inhaled racemic epinephrine and the worsening of her respiratory symptoms, a fiber optic examination was conducted.

**Figure 3:**
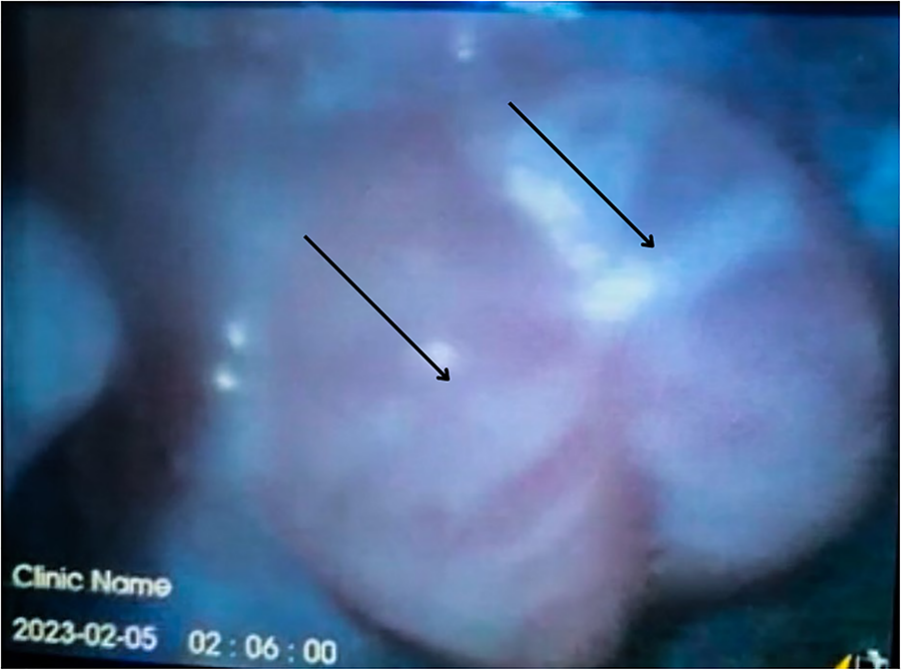
Two years old male with thermal injury-induced epiglottitis. Fiberoptic examination showing severe edema of the epiglottis with near-complete obstruction of the vocal cords.

This examination revealed severe swelling of the epiglottic structure, circumferential burn, and near-complete obstruction of the vocal cords, indicative of traumatic epiglottitis (Figure 3). Due to the extensive upper airway involvement and the difficulty of endotracheal tube insertion, a tracheostomy was performed. After 4 days of intubation and treatment with IV dexamethasone, a significant improvement was observed. No burns, scars, or swelling were noted on the repeat direct laryngoscopy. The child was successfully extubated and discharged 2 days later, following a normal fiber optic endoscopy. Upon discharge, the only remaining findings were a swollen and erythematous soft palate and uvula. After 3 months of follow-up, the patient is now fully recovered.

### Case two

A previously healthy 2-year-old male was brought to the emergency department with acute symptoms of drooling, dysphagia, and stridor (Figure 1). His mother reported an incident where he accidentally sprayed an alkaline solution into his mouth. At presentation, the patient appeared irritable, with stridulous breathing and a forward-leaning position. His vital signs included a normal temperature, elevated respiratory rate, heart rate, and normal blood pressure. The case had completely received the Hib vaccination at 16 months.

**Figure 4:**
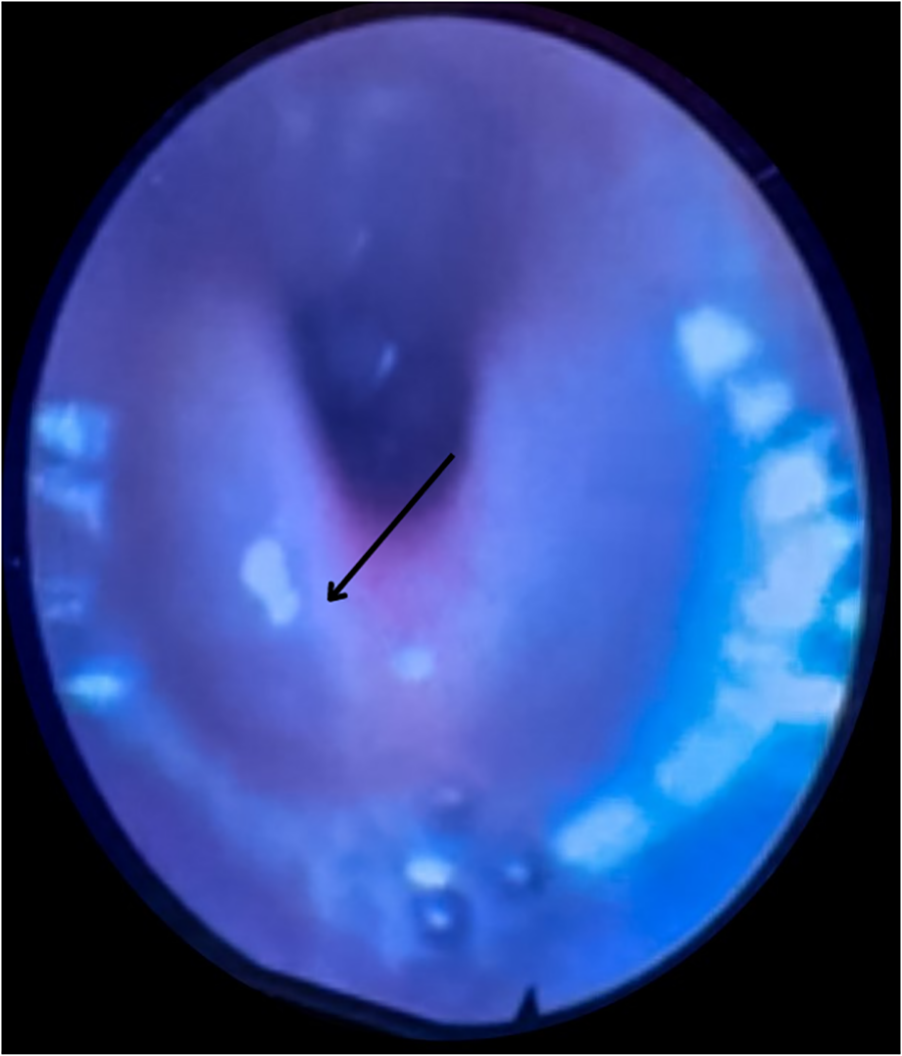
Four years old male with chemical exposure epiglottitis. Fiberoptic examination showing swollen and erythematous supraglottic structures with partial obstruction.

Examination of his mouth and oral pharynx revealed whitish plaques on the soft palate and edema of the soft palate and oropharynx. The patient's hemogram showed normal results. A fiber optic examination demonstrated swollen and erythematous supraglottic structures with partial obstruction (Figure 4).

Immediate airway management was initiated with the placement of an endotracheal tube. He was treated with four intravenous boluses of dexamethasone and penicillin. Although a blood culture taken prior to admission was negative, antibiotics were administered. After 3 days of intubation, a direct laryngoscopy showed notable improvement with no residual edema. The patient was extubated and discharged from the hospital 2 days later following a normal fiber optic endoscopy. The patient was followed-up for 1 month and was fully recovered upon examination.

## Discussion

Epiglottitis, while uncommon, presents a critical emergency situation. The widespread implementation of the Hib vaccine for infants has led to a dramatic decrease in cases of infectious epiglottitis. Presently, the causes of epiglottitis are predominantly non-infectious. Traumatic supraglottitis can result from various types of airway injuries, including mechanical, thermal, or chemical (7, 8). The airways in children are comparatively narrower than those in adults (6). Consequently, any neck or upper airway injury can result in significant swelling and more rapidly compromise the airway in young patients.

This paper focuses on the traumatic variants of non-infectious epiglottitis, an area not comprehensively addressed in existing literature. Currently, there are no definitive guidelines for treating traumatic epiglottitis. To address this gap, we have undertaken a literature review and developed a multidisciplinary management approach for this type of epiglottitis. The presented cases, along with the findings from the literature review (Supplementary Table S1), provide valuable insights into the diverse etiology, presentation, and management of non-infectious epiglottitis in children. The review of 23 cases highlighted that while thermal injuries and foreign body ingestions are common causes (56.51%), a variety of other factors such as caustic injuries and trauma can also lead to this condition. The mean age of presentation was approximately 46 months, with a predominance in males (73.91%). Commonly noted symptoms were stridor (56.52%), drooling (56.52%), dysphagia (47.82%), and dyspnea (43.47%), similar to our cases.

In the second cases, antibiotic therapy was initiated due to the presumptive diagnosis of Hib epiglottitis, utilizing penicillin. However, considering the beta-lactamase production capabilities of many *H. influenzae type B* strains, penicillin may not be the optimal choice (9). Based on current local and global antibiotic resistance patterns, ampicillin or, more reliably, ceftriaxone would be preferred due to their efficacy against beta-lactamase-producing strains (3). This selection underscores the importance of antibiotic stewardship and the need for up-to-date knowledge on local pathogen resistance patterns, ensuring that management choices align with the best chance for successful treatment outcomes.

The management strategies varied, with over half of the cases in the literature review receiving antibiotics and steroids, and about 40% receiving epinephrine. This aligns with the treatments provided in our cases, where steroids played a crucial role in the management. Airway intervention was necessary in 82.60% of cases, emphasizing the critical nature of early diagnosis and intervention (Supplementary Table S2). However, intubation was delayed in 57.89% of intubated cases. ICU admission was required in 56.52% of cases, with a mean ICU and hospital stay of 4.78 (SD = 1.86) and 5.88 (3.01) days, respectively. Importantly, complications occurred in 47.82% of them with two deaths occurring (10).

The role of vaccination, particularly against *Hemophilus influenza type B*, in the context of non-infectious epiglottitis is intriguing (11). While only a small proportion of cases in the literature review were vaccinated, it raises questions about the potential impact of vaccination status on the presentation, management, and outcomes of these patients. Vaccination might influence the clinical suspicion towards non-infectious causes and could potentially alter the approach to management and airway intervention. In our review, we noted that among the five vaccinated cases, 80% required intubation (delayed; mean = 1.6 days) and ICU admission. Vaccinated patients had similar ICU and hospital length of stay to that of the whole population. One case required readmission with no deaths being reported. More research is needed to understand the full implications of vaccination status in these cases.

Finally, the recognition of non-infectious causes in pediatric epiglottitis is crucial for appropriate management. These case reports, supported by a comprehensive literature review, highlight the need for heightened awareness and consideration of non-infectious causes in pediatric epiglottitis. They also underscore the importance of individualized patient care and the potential role of vaccination in influencing clinical outcomes.

## Data Availability

The original contributions presented in the study are included in the article/**Supplementary Material**, further inquiries can be directed to the corresponding author.

## Supplementary material

Supplementary Figure S1 The anatomical differences in the airways of adults and children.
