# Supplementary material for: The emergence of non-infectious epiglottitis after the era of Hemophilus influenza type B universal vaccination: two case reports and literature review
Source: Front Pediatr. 2024 Jul 4;12:1374311. doi: 10.3389/fped.2024.1374311 (PMC11254710; doi:10.3389/fped.2024.1374311)
Supplement: Supplementary file 1 [file Table1.docx]

**Supplementary Table 1.** The clinicodemographic characteristics of pediatric cases with non-infectious epiglottitis reported in the literature from 1989 to 2023

| Author (YOP) | Age | Gender | Presenting Symptoms | Etiology | H. influenza Vaccination | Medical Treatment | | | |
| --- | --- | --- | --- | --- | --- | --- | --- | --- | --- |
|  |  |  |  |  |  | Antibiotic | Steroid | Epinephrine | Others |
| Alkaabi (2023) (8) | 27 months | Male | Stridor - dyspnea - cyanosis | Spilled hot coffee | - | Broad-spectrum antibiotics | Budesonide 2g | Nebulized epinephrine at 0.25mL | - |
| Bozzella (2020) (9) | 15 years | Female | Dysphagia - Hoarseness of voice - regurgitation - food avoidance | Using a JUUL (e-cigarette) | Vaccinated | Ampicillin | Dexamethasone | Nebulized epinephrine | Sulbactam |
| Harjacek (1992) (10) | 3 years | Male | Stridor - dysphagia - hoarseness of voice - air gasping - subcostal retraction | Swallowing hot tea | - | Ceftriaxone | - | Nebulized epinephrine | - |
| Inaguma (2019) (3) | 18 months | Male | Stridor - dysphagia | Hot food swallowing | Vaccinated | Cefotaxime | Dexamethasone | Nebulized epinephrine | - |
| Kabbani (1995) (11) | 8 months | Female | Stridor - drooling - dyspnea | Traumatic (Blind finger sweep to remove a pharyngeal foreign body) | - | Cefotaxime + Clindamycin | - | Nebulized epinephrine | - |
| Kavanagh (2008) (4) | 15 months | Male | Stridor - drooling - dysphagia - restlessness | Swallowing a bottle cap | - | Cefuroxime | Yes (NC | - | - |
| Kudchadkar (2014) (12) | 22 months | Male | Stridor - drooling | Steam blast and a spill of boiling water | - | - | Yes (NC) | - | Beta-agonists |
| Lai (2000) (13) | 17 months | Male | Stridor - drooling - dysphagia - hoarseness of voice | Acetic anhydride solution was given to lyse an impacting fishbone in the esophagus | - | Penicillin | Dexamethasone | Nebulized epinephrine | - |
|  | 19 months | Female | Stridor - drooling - dyspnea | Swallowing hot water | - | Penicillin | Dexamethasone | Nebulized epinephrine | - |
| Lichtor (2016) (2) | 24 months | Female | Drooling - dysphagia | Foreign body | - | Vancomycin + ceftriaxone + cefdinir | - | - | - |
| O'Bier (2005) (14) | 12 years | Male | Dyspnea - sore throat - angioedema | Hereditary angioedema | Vaccinated | - | - | Subcutaneous & nebulized epinephrine | Diphenhydramine, ami-caproic acid (Amicar) |
| Parsons (1996) (15) | 6 years | Male | Stridor | Trauma from either the anesthesiologist’s intubation or the otolaryngologist’s Crow Davismouth gag blade | - | - | - | - | - |
|  | 8 months | Male | Stridor - drooling - dysphagia | Ingestion of water bed tablet (highly alkaline product) | - | - | - | - | - |
| Rao (2021) (16) | 17 years | Male | Drooling - dysphagia - hoarseness of voice - odynophagia - fever | Cannabis induced thermal injury | Vaccinated | IV vancomycin + ceftriaxone then oral amoxicillin / clavulanate | Dexamethasone | - | ketorolac |
| Verhees (2018) (17) | 15 months | Male | Stridor - dyspnea - burns | Submersion by just-boiled milk | - | - | - | - | IV analgesics |
| Yokoyama (2018) (18) | 13 months | Female | Stridor - drooling - irritability - sternal retraction | Forcefully opening mouth and placing fingers in the child’s mouth during convulsions to prevent biting of tongue | Vaccinated | Yes (NC) | Yes (NC) | Yes (NC) | - |
| Bonadio (1991) (6) | 6 years | Male | Dyspnea - dysphagia - sore throat - cyanosis | - | - | - | - | - | - |
|  | 9 months | Male | Dyspnea - cyanosis | - | - | - | - | - | - |
|  | 9 years | Female | Dyspnea - dysphagia - cyanosis - fever | - | - | - | - | - | - |
| Deutsch (2004) (19) | 9 months | Male | Dysphagia - bleeding - tachypnea | Blind finger sweeping of foreign body in pharynx | - | Yes (NC) | Yes (NC) | - | - |
| Yen (2003) (20) | 18 months | Male | Stridor - drooling - irritability - sternal retraction | Blind finger sweeping of coin in pharynx | - | - | Yes (NC) | - | - |
| Laufkotter (1989) (21) | 3 months | Male | Drooling - dysphagia - irritability - angioedema - fever | Hot vegetables | - | Yes (NC) | Yes (NC) | - | - |
| Watts (1996) (22) | 16 months | Male | Drooling - dyspnea - irritability - dry cough | Hot water on face and chest | - | - | - | - | - |

YOP: year of publication; IV: intravenous; NC: not clarified

**Supplementary Table 2.** The management and clinical outcomes of pediatric cases with non-infectious epiglottitis reported in the literature from 1989 to 2023

| Author (YOP) | Airway Intubation | | | | | Outcomes | | | | | |
| --- | --- | --- | --- | --- | --- | --- | --- | --- | --- | --- | --- |
|  | Intubation | Immediate | Delayed | Tracheostomy | Intubation (days) | ICU Admission | ICU (days) | LOS (days) | Complications | Readmission | Death |
| Alkaabi (2023) (8) | Yes | Yes | - | - | - | Yes | 5 | - | moderate to severe respiratory distress | - | - |
| Bozzella (2020) (9) | - | - | - | - | - | Yes | - | 7 | - | Yes | - |
| Harjacek (1992) (10) | Yes | - | Yes | - | 1 | - | - | 3 | Respiratory distress | - | - |
| Inaguma (2019) (3) | Yes | - | Yes | - | 1 | - | - | 3 | - | - | - |
| Kabbani (1995) (11) | Yes | - | Yes | - | - | Yes | 2.5 | 7 | Respiratory distress | - | - |
| Kavanagh (2008) (4) | Yes | - | Yes | - | 4 | Yes | 5 | 6 | - | - | - |
| Kudchadkar (2014) (12) | Yes | - | Yes | Yes | 28 | Yes | - | - | - | - | - |
| Lai (2000) (13) | - | - | - | - | - | - | - | - | Respiratory distress | - | - |
|  | Yes | - |  | - | 3 | - | - | - | - | - | - |
| Lichtor (2016) (2) | Yes | - | Yes | - | 4 | Yes | 7 | 7 | - | - | - |
| O'Bier (2005) (14) | Yes | - | Yes | - | 1 | Yes | 2 | 2 | Respiratory distress | - | - |
| Parsons (1996) (15) | - | - | - | - | - | - | - | - | - | - | - |
|  | - | - | - | - | - | Yes | - | - | - | - | - |
| Rao (2021) (16) | Yes | - | Yes | - | 2 | Yes | - | - | - | - | - |
| Verhees (2018) (17) | Yes | Yes | Yes | - | 8 | Yes | - | - | Respiratory distress | - | - |
| Yokoyama (2018) (18) | Yes | - | Yes | - | 4 | Yes | 6 | 6 | Moderate respiratory distress | - | - |
| Bonadio (1991) (6) | Yes | Yes | - | - | 2.3 | - | - | - | Pulmonary edema | - | - |
|  | Yes | Yes | - | - | 0.5 | - | - | - | Pulmonary edema, respiratory distress | - | Yes |
|  | Yes | Yes | - | - | - | - | - | - | Pulmonary edema, respiratory distress | - | Yes |
| Deutsch (2004) (19) | Yes | - | - | - | 1.5 | - | - | - | - | - | - |
| Yen (2003) (20) | Yes | - | Yes | - | 0.5 | - | - | - | Mild to moderate respiratory distress | - | - |
| Laufkotter (1989) (21) | Yes | - | - | - | 2 | Yes | - | - | - | - | - |
| Watts (1996) (22) | Yes | - | - | - | 4 | Yes | 6 | 12 | - | - | - |

YOP: year of publication
