# Supplementary figures and images for: The emergence of non-infectious epiglottitis after the era of Hemophilus influenza type B universal vaccination: two case reports and literature review
Source: Front Pediatr. 2024 Jul 4;12:1374311. doi: 10.3389/fped.2024.1374311 (PMC11254710; doi:10.3389/fped.2024.1374311)

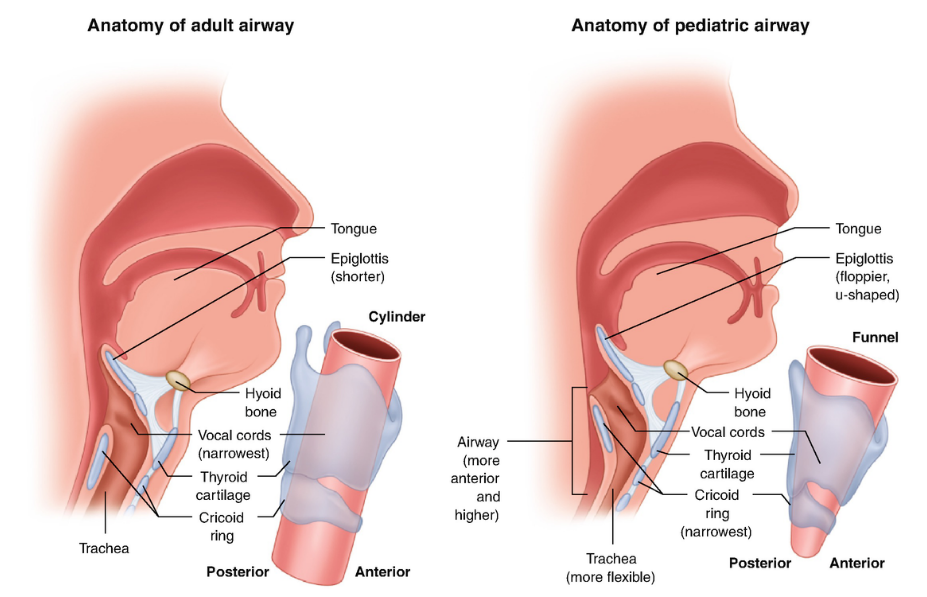

Supplement: Supplementary Figure S1 — The anatomical differences in the airways of adults and children. [file Image1.tif]
